# Impact of Fenestration Size and Prosthesis Diameter on Hearing Outcomes After Stapedotomy: A Review

**DOI:** 10.7759/cureus.89692

**Published:** 2025-08-09

**Authors:** Shrikrishna B H, Deepa G

**Affiliations:** 1 Otorhinolaryngology Head-Neck Surgery, All India Institute of Medical Sciences, Bibinagar, Hyderabad, IND; 2 Anatomy, All India Institute of Medical Sciences, Bibinagar, Hyderabad, IND

**Keywords:** air-bone gap, hearing loss, otosclerosis, prosthesis design, stapedotomy

## Abstract

This systematic review investigates the influence of fenestration size and prosthesis diameter on hearing outcomes in patients undergoing primary stapedotomy for otosclerosis. A total of 11 studies were included, comprising randomized controlled trials, cohort studies, and one cross-sectional study, with follow-up durations ranging from three months to one year. Fenestration sizes most commonly ranged from 0.5 mm to 0.8 mm, while prosthesis diameters varied between 0.4 mm and 0.6 mm. Across studies, postoperative air conduction thresholds improved by 20-30 dB, and air-bone gap (ABG) closure within 10 dB was achieved in 57% to 88.9% of cases. Although some studies reported marginally better bone conduction gains with 0.6 mm prostheses, no definitive audiological superiority was established for any specific fenestration-prosthesis size combination. The use of larger prosthesis diameters was associated with modest improvements in mid-frequency sound transmission, particularly at 2000 Hz, but these differences were often not statistically significant. Postoperative complications were minimal, with transient vertigo being the most commonly reported, especially in laser-assisted techniques. Importantly, no study linked complication rates directly to specific fenestration or prosthesis sizes. Overall, the findings suggest that both fenestration size and prosthesis diameter within the commonly used ranges yield consistently favorable audiological outcomes without significantly affecting safety. The choice of prosthesis size may be tailored to individual anatomical and surgical considerations without compromising efficacy. This review highlights the flexibility in surgical approach for stapedotomy, supporting the use of varying size parameters according to intraoperative conditions and surgeon preference.

## Introduction and background

Otosclerosis is a progressive disease causing abnormal bone growth in the middle ear, leading to conductive hearing loss. Stapedotomy is a microsurgical procedure that restores hearing by creating a small hole (fenestration) in the stapes footplate and inserting a prosthesis, thereby reestablishing sound transmission to the inner ear. Stapedotomy is a commonly performed surgical intervention for otosclerosis. It entails making a fenestration in the stapes footplate and inserting a prosthesis to restore ossicular chain integrity. Despite numerous advancements in surgical technique and prosthesis design, the ideal fenestration size and prosthesis diameter for achieving maximal hearing restoration are still up for debate [1,2].

Previous studies have investigated various surgical parameters, including the size of the fenestration and the material, type, and dimensions of the prosthesis, aiming to enhance the procedure's audiological outcomes [3]. While some surgeons support smaller fenestrations to reduce trauma to the inner ear, others suggest that larger fenestrations allow better prosthesis coupling and possibly enhanced sound transmission [4,5]. Likewise, prosthesis diameter may influence the transmission of acoustic energy, with low-frequency hearing gains and cochlear safety [6,7].

Some trials report superior outcomes with specific combinations, whereas others suggest insignificant differences [8]. Additionally, complications such as transient vertigo and inner ear trauma have raised worries over technique-specific risks [9]. The current review creates available evidence to evaluate the audiometric outcomes and safety profiles associated with different fenestration sizes and prosthesis diameters in primary stapedotomy for otosclerosis.

## Review

Methods

Eligibility Criteria

We included studies that focused exclusively on patients with otosclerosis undergoing primary stapedotomy. The studies had to give the fenestration size and prosthesis diameter in millimeters, as well as the results of the patients' hearing after surgery, measured by normal audiometric methods like air-bone gap (ABG) or pure-tone averages. Only randomized controlled trials, prospective cohort studies, and retrospective cohort studies with at least 10 patients and a follow-up duration of at least three months were considered. Studies with concurrent middle ear pathologies or without clearly distinguishable stapedotomy outcomes were excluded.

Search Strategy

A comprehensive literature search was conducted in PubMed using the following Boolean search strategy: ((stapedotomy[Title/Abstract]) AND (fenestration size[Title/Abstract])) AND (piston diameter[Title/Abstract])) OR (prosthesis size[Title/Abstract])) OR (piston size[Title/Abstract])) AND (hearing outcome[Title/Abstract])) OR (hearing gain[Title/Abstract])) OR (air-bone gap[Title/Abstract])) OR (audiological outcome[Title/Abstract])). The initial search yielded 3,193 results. Automatic filters were applied to restrict results to studies published in the last 10 years, available as free full text, written in English, involving human participants, and classified as randomized clinical trials (RCTs), resulting in 18 records. Following application of predefined screening criteria, seven studies were excluded, and 11 studies were included in the final review. Preprints were also excluded from the selection (Table 1).

**Table 1:** Search strategy Detailed search strategy used for identifying eligible studies, including databases searched, search terms and filters applied.

Database	Search Strategy	Initial Results	Filters Applied (Automatic)	Results After Filters	Excluded During Screening	Included in Final Review
PubMed	((stapedotomy[Title/Abstract]) AND (fenestration size[Title/Abstract])) AND (piston diameter[Title/Abstract])) OR (prosthesis size[Title/Abstract])) OR (piston size[Title/Abstract])) AND (hearing outcome[Title/Abstract])) OR (hearing gain[Title/Abstract])) OR (air-bone gap[Title/Abstract])) OR (audiological outcome[Title/Abstract]))	3,193	Published in the last 10 years- Free full text available- English language- Human participants- Study type: Randomized Clinical Trials (RCTs)- Preprints excluded	18	7	11

Selection Process

Eighteen studies were included based on holistic judgment, considering the fulfillment of all listed inclusion criteria. A dual independent review was performed, and disagreements were resolved by consensus.

Data Collection

**Figure 1:**
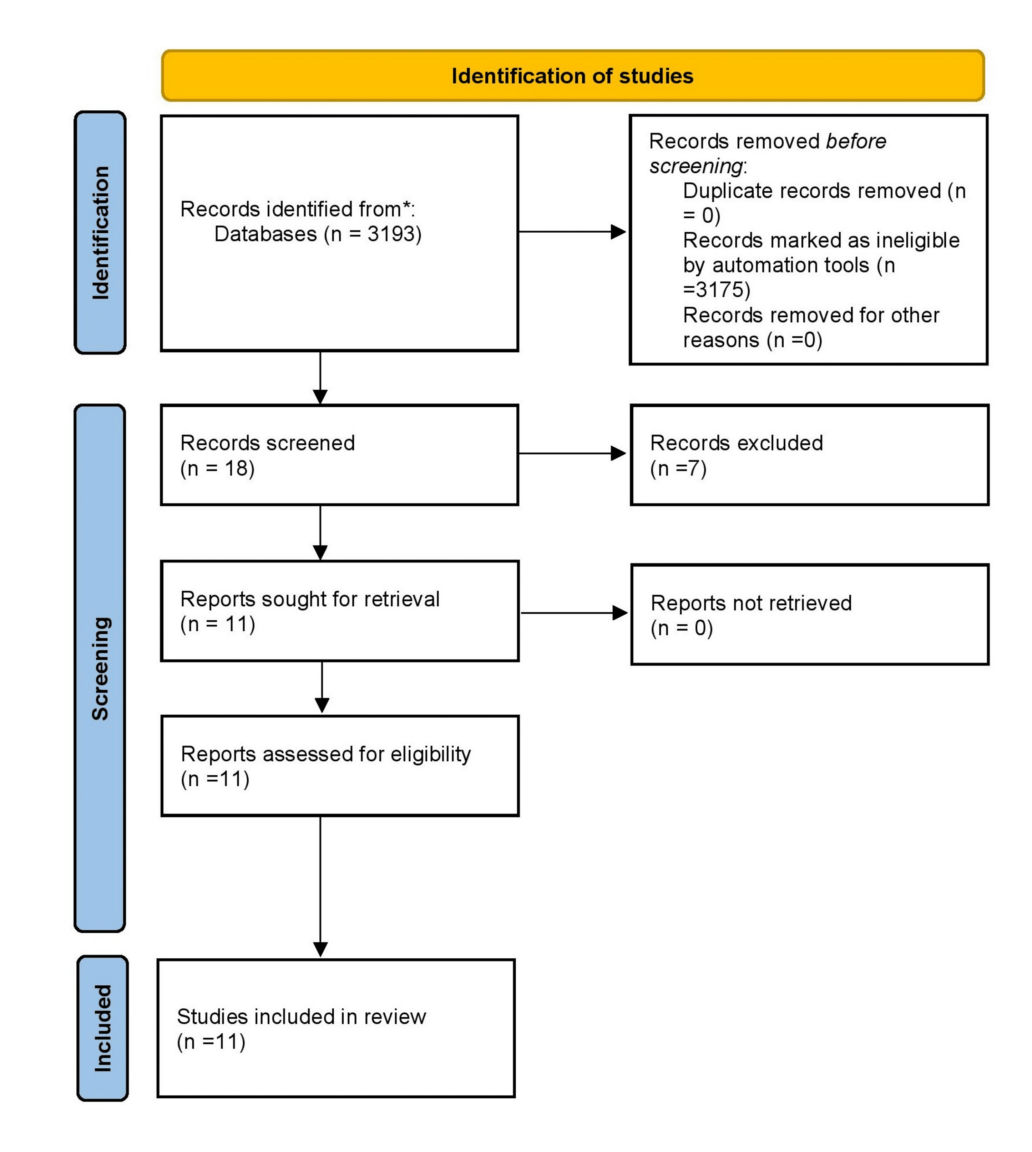
PRISMA flow chart PRISMA: Preferred Reporting Items for Systematic Reviews and Meta-Analyses

Data were extracted using structured prompts for: study design, participant demographics, fenestration size, prosthesis diameter and material, follow-up duration, and hearing outcomes (AC, BC, ABG, success rates) (Figure 1). Extracted data were verified manually against study content where needed (Table 2). A total of 18 papers were screened, of which seven papers were excluded based on screening criteria.

**Table 2:** Data extraction table Summary of included studies with study design, participant details, fenestration size, prosthesis diameter and material, and follow-up duration.

Author	Study Design	Participants	Fenestration Size	Prosthesis Diameter	Prosthesis Material	Follow-up
Faranesh et al., 2017 [1]	RCT	18	0.5 mm / 0.7 mm	0.4 / 0.6 mm	Fluoroplastic	12 months
Hervochon et al., 2021 [2]	Retrospective	175 (320 ears)	Not Reported	0.6 mm	Teflon	3 mo, 1 yr
Velusamy et al., 2021 [3]	Prospective	70	0.8 mm	0.4 mm	Nitinol	3 months
Faramarzi et al., 2020 [4]	RCT	170	0.7 mm	0.5 / 0.6 mm	Teflon / Titanium	6 months
Bagger-sjöbäck et al., 2015 [5]	RCT	156	Not Reported	0.4 mm	Teflon / Platinum	1 year
Hamerschmidt et al., 2017 [6]	Cross-sectional	12	Not Reported	Not Reported	Not Reported	3 months
Karaca et al., 2016 [7]	RCT	69	Not Reported	0.6 mm	Teflon	Not Mentioned
Moneir et al., 2023 [8]	RCT	60	0.7 mm	0.6 mm	Teflon	3 months
Kumar et al., 2022 [9]	Retrospective	74	Not Reported	Not Reported	Not Reported	3, 6 months
Kim et al., 2022 [10]	Retrospective	583 (18 with otosclerosis)	Not Reported	Not Reported	Not Reported	6 months
Barati et al., 2024 [11]	RCT	72	Not Reported	Not Reported	Not Reported	Not Mentioned

Risk of bias assessment

Risk of bias was assessed using the ROB2 tool for randomized trials and the Newcastle-Ottawa Scale (NOS) for observational studies. Parameters considered included selection bias, measurement of outcomes, and adequacy of follow-up (Table 3).

**Table 3:** Risk of bias summary Risk of bias assessment for included studies using ROB2 for randomized trials and Newcastle–Ottawa Scale for observational studies.

Author	Design	Risk of Bias
Faranesh et al., 2017 [1]	RCT	Low
Hervochon et al., 2021 [2]	Retrospective	Moderate
Velusamy et al., 2021 [3]	Prospective	Low
Roosta et al., n.d. [4]	RCT	Low
Bagger-sjöbäck et al., 2015 [5]	RCT	Low
Hamerschmidt et al., 2017 [6]	Cross-sectional	High
Karaca et al., n.d. [7]	RCT	Moderate
Moneir et al., 2023 [8]	RCT	Low
Kumar et al., 2022 [9]	Retrospective	Moderate
Kim et al., 2022 [10]	Retrospective	Moderate
Barati et al., 2024 [11]	RCT	Low

Data synthesis

Due to clinical heterogeneity and variability in the reporting of audiometric outcomes, meta-analysis was not performed. A qualitative synthesis of results was conducted, and findings were organized according to fenestration size, prosthesis diameter, and associated hearing outcomes.

Results

Data were extracted using structured prompts for: study design, participant demographics, fenestration size, prosthesis diameter and material, follow-up duration, and hearing outcomes (AC, BC, ABG, success rates) (Figure 1). Extracted data were verified manually against study content where needed (Table 2). A total of 18 papers were screened, of which seven papers were excluded based on screening criteria [11-16]. Risk of bias was assessed using the ROB2 tool for randomized trials and the Newcastle-Ottawa Scale (NOS) for observational studies. Parameters considered included selection bias, measurement of outcomes, and adequacy of follow-up (Table 3). The included studies reflect diverse methodological designs, with six randomized controlled trials, three retrospective cohorts, one prospective cohort, and one cross-sectional study. Only four studies reported fenestration size explicitly, ranging from 0.5 mm to 0.8 mm. Prosthesis diameter was noted in seven studies, typically between 0.4 mm and 0.6 mm. Teflon was the most common material, followed by fluoroplastic and nitinol.

Discussion

Regarding fenestration size, only four studies explicitly reported this parameter [1,3,4,8]. The fenestration sizes ranged from 0.5 mm to 0.8 mm. Studies such as Faranesh et al. [1] and Moneir et al. [8] demonstrated air-bone gap (ABG) closure within 10 dB in 76.7-88.9% of cases. Velusamy et al. [3] observed ABG closure in 80-85.7% of cases with a 0.8-mm fenestration, suggesting favorable outcomes even at the larger end of the range. However, these improvements did not differ significantly between groups, indicating that a wide range of fenestration sizes is clinically acceptable.

In terms of prosthesis diameter, the most frequently used sizes were 0.4 mm and 0.6 mm. Comparative studies such as those by Faranesh et al. [1] and Roosta et al. [4] found similar improvements in ABG closure and air conduction thresholds across different prosthesis sizes. Notably, Faranesh et al. [1] reported a marginally better bone conduction gain with 0.6 mm prostheses, although the clinical significance of this difference remains limited. These findings suggest that a prosthesis diameter between 0.4 and 0.6 mm does not substantially affect hearing outcomes. Studies that failed to report prosthesis size still demonstrated meaningful hearing gains, further emphasizing the minimal impact of this variable [2,6,9-11].

Postoperative complications were generally low across studies. Transient vertigo was the most commonly reported adverse event and was notably more frequent in laser-assisted procedures, such as those reported by Kumar et al. [9]. However, no study linked vestibular symptoms directly to fenestration size or prosthesis diameter. Only one study [3] documented a minor worsening in bone conduction in a subset of patients (14-17%), but again, this was not associated with prosthesis specifications. These findings align with the current understanding that modern stapedotomy techniques are safe, with a low risk of serious inner ear damage.

Long-term follow-up data, where available, demonstrated stable hearing results. Bagger-sjöbäck et al. [5] and Hervochon et al. [2] followed patients for up to one year and noted no deterioration in audiometric outcomes or increased complication rates. This suggests that both small and large fenestration sizes, as well as various prosthesis diameters, provide lasting functional improvement.

Research consistently shows that both fenestration size and prosthesis diameter influence hearing outcomes after stapedotomy, though the effects are nuanced. Meta-analyses and large cohort studies indicate that using a larger prosthesis diameter (typically 0.6 mm) is associated with slightly better postoperative air conduction thresholds, smaller air-bone gaps, and higher rates of successful hearing restoration compared to smaller diameters (0.4 mm), especially at speech frequencies and lower frequencies, without increasing the risk of sensorineural hearing loss [17-19]. Experimental models and clinical studies suggest that larger pistons improve sound transmission, with round window velocities increasing by 2-4 dB as piston diameter increases from 0.4 mm to 0.8 mm, particularly at midrange frequencies [20,21]. However, some studies find the differences between 0.4 mm and 0.6 mm prostheses to be modest and not always statistically significant, and overall hearing outcomes remain excellent with both sizes [22]. The combination of a small fenestra and a larger-diameter piston appears to offer the best balance of safety and hearing improvement, though surgical conditions and individual anatomy may dictate the final choice. Otoacoustic emission testing has not shown clear clinical value in predicting hearing outcomes post-stapedotomy [23].

The diameter of the prosthesis used in stapedotomy has a measurable impact on hearing outcomes, though the differences are often modest. Meta-analyses and large retrospective studies show that a 0.6 mm piston generally provides better postoperative air conduction thresholds, smaller air-bone gaps, and higher rates of successful hearing restoration compared to a 0.4 mm piston, with success rates of 81.1% versus 75.1% and better results, particularly at speech frequencies [24]. Experimental models confirm that larger pistons (up to 0.8 mm) further improve sound transmission, especially at lower and midrange frequencies, though the effect is relatively small (2-4 dB improvement). However, several clinical studies report that overall hearing gains, pure tone averages, and air-bone gap closures are similar between 0.4 mm and 0.6 mm prostheses, with only minor advantages for the larger size at specific frequencies such as 2000 Hz [1,20]. The choice of prosthesis diameter should also consider the anatomical variability of the long process of the incus, as a secure fit is crucial for optimal outcomes [25].

The diameter of a prosthesis can significantly affect clinical outcomes, particularly in stapedotomy for otosclerosis. Meta-analyses and large pooled studies show that a 0.6-mm stapes prosthesis yields better hearing results than a 0.4-mm prosthesis, with higher rates of air-bone gap closure within 10 dB (81.1% vs. 75.1%), improved postoperative air conduction thresholds, and greater overall success rates [26].

Several limitations in the reviewed studies should be acknowledged. First, only a subset of studies provided detailed technical parameters, limiting direct comparison across all data. The heterogeneity in surgical techniques (e.g., use of laser vs. manual perforation), prosthesis materials, and postoperative audiometric measures precluded meta-analysis. Additionally, many studies lacked blinding and randomization, introducing potential biases. The review's strength lies in its strict inclusion criteria, which ensured that only studies that looked at fenestration size, prosthesis diameter, and audiometric results in primary stapedotomy were included.

## Conclusions

Stapedotomy offers consistent audiological benefits regardless of fenestration size or prosthesis diameter within standard ranges. Prosthesis choice can be tailored to anatomical and intraoperative considerations. Clinically, this supports flexibility in surgical planning. Future research should explore long-term outcomes and refine prosthesis design for enhanced frequency-specific hearing restoration.
